# CT colonography: size reduction of submerged colorectal polyps due to electronic cleansing and CT-window settings

**DOI:** 10.1007/s00330-018-5416-0

**Published:** 2018-05-14

**Authors:** Christian Bräuer, Philippe Lefere, Stefaan Gryspeerdt, Helmut Ringl, Ali Al-Mukhtar, Paul Apfaltrer, Dominik Berzaczy, Barbara Füger, Julia Furtner, Christina Müller-Mang, Matthias Pones, Martina Scharitzer, Ramona Woitek, Anno Graser, Michael Weber, Thomas Mang

**Affiliations:** 1 Department of Biomedical Imaging and Image-guided Therapy, Medical University of Vienna, Währinger Gürtel 18–20, 1090 Vienna, Austria; 2 AZ Delta, Department of Radiology, Bruggesteenweg 90, 8800 Roeselare, Belgium; 3 Institut für CT und MRT Gänserndorf, Umfahrungsstrasse Nord 7, 2230 Gänserndorf, Austria; 4 Radiologie München, Burgstrasse 7, 80331 Munich, Germany

**Keywords:** CT colonography, Virtual colonoscopy, Neoplasms, colorectal, Colonic polyps, Cancer screening

## Abstract

**Objectives:**

To assess whether electronic cleansing (EC) of tagged residue and different computed tomography (CT) windows influence the size of colorectal polyps in CT colonography (CTC).

**Methods:**

A database of 894 colonoscopy-validated CTC datasets of a low-prevalence cohort was retrospectively reviewed to identify patients with polyps ≥6 mm that were entirely submerged in tagged residue. Ten radiologists independently measured the largest diameter of each polyp, two-dimensionally, before and after EC in colon, bone, and soft-tissue-windows, in randomised order. Differences in size and polyp count before and after EC were calculated for size categories ≥6 mm and ≥10 mm. Statistical testing involved 95% confidence interval, intraclass correlation and mixed-model ANOVA.

**Results:**

Thirty-seven patients with 48 polyps were included. Mean polyp size before EC was 9.8 mm in colon, 9.9 mm in bone and 8.2 mm in soft-tissue windows. After EC, the mean polyp size decreased significantly to 9.4 mm in colon, 9.1 mm in bone and 7.1 mm in soft-tissue windows. Compared to unsubtracted colon windows, EC, performed in colon, bone and soft-tissue windows, led to a shift of 6 (12,5%), 10 (20.8%) and 25 (52.1%) polyps ≥6 mm into the next smaller size category, thus affecting patient risk stratification.

**Conclusions:**

EC and narrow CT windows significantly reduce the size of polyps submerged in tagged residue. Polyp measurements should be performed in unsubtracted colon windows.

**Key Points:**

*• EC significantly reduces the size of polyps submerged in tagged residue.*

*• Abdominal CT-window settings significantly underestimate 2D sizes of submerged polyps.*

*• Size reduction in EC is significantly greater in narrow than wide windows.*

*• Underestimation of polyp size due to EC may lead to inadequate treatment.*

*• Polyp measurements should be performed in unsubtracted images using a colon window.*

**Electronic supplementary material:**

The online version of this article (10.1007/s00330-018-5416-0) contains supplementary material, which is available to authorized users.

## Introduction

Computed tomography colonography (CTC) is the radiological examination of choice for the diagnosis of colorectal neoplasia [1].

The evaluation of CTC examinations may be limited by residual stool and fluid, and thus either simulating or obscuring polypoid colonic lesions. By oral administration of positive contrast media, residual faecal material is “tagged”, i.e. rendered hyperdense. Soft-tissue lesions submerged within hyperdense tagged faecal residue can be depicted on 2D images. Faecal tagging has been shown to increase the sensitivity as well as the specificity of CTC and is now considered a mandatory part of bowel preparation [2, 3]. However, colonic mucosa that is obscured by tagged faecal residue is not accessible to endoluminal three-dimensional (3D) views and requires additional time-consuming two-dimensional (2D) evaluation. An approach to overcome this limitation includes “electronic cleansing” (EC) software algorithms designed to digitally subtract tagged faecal residue from a CTC dataset. In fact, EC has been shown to improve colonic evaluation and polyp detection [4–6]. However, it has not been determined in vivo*,* as yet, whether EC affects the size of polyps that are submerged under tagged faecal residue. Related size differences may influence clinical reporting of polyps and, thus, patient treatment.

The purpose of this retrospective study was to assess whether electronic cleansing of tagged residue and different CT-window settings influence the size of colorectal polyps in CTC studies and whether related size differences lead to different grading into clinically applied size categorisations.

## Materials and methods

### Patient population

The contributing institutions had performed the CTC examinations with written, informed consent under approval of their local Institutional Review Board. The protocol of this retrospective study was approved by the Institutional Review Board (IRB) at the Medical University of Vienna; written, informed consent was waived.

The CTC image data within this database were acquired as a part of three IRB-approved prospective screening trials with the aim of assessing the performance of CTC compared to optical colonoscopy (OC) to screen patients at average risk for colorectal cancer [7–9]. Anonymised patient data accrued originally during the screening study described by Pickhardt et al. [8] were downloaded from a publicly accessible CTC training web site [10].

In all patients, the indication for CTC was to screen for colorectal cancer and polyps. All patients underwent CTC and subsequent OC with a histopathological workup. Patient datasets were anonymised at the respective institutions that had performed the CTC exams.

The sample size calculation indicated that 900 patients were required to identify 38 polyps ≥6 mm and submerged in tagged faecal residue to power the study accordingly. It is further described in the “Electronic supplementary material” (ESM 1)*.*

### Examination technique

CTC examination techniques for the included patient datasets performed in adherence to published standards [3, 11]. They were described previously in detail [7–9] and are summarised in the ESM 1.

### Reference standard

OC and histological reports served as the standard of reference for the presence of colorectal polyps. Abstracted report files from CTC, OC and from the histological evaluation were available from two sources [7, 10]. The other case-contributing site provided the information from the CTC/OC correlation trial in an Excel sheet, containing the anonymised patient data and the reported findings of CTC, OC and lesion histology [9]. Thus, polyp size and shape information, as well as segmental location and polyp histology, were available for each patient. Complete access to these reference data was used to perform a directed search in the database to identify those patients with submerged colorectal lesions measuring ≥6 mm at CTC, detected with both CTC and OC. A polyp was classified as submerged if it was completely submerged under residual tagged fluid with a density of at least 100 HU, at least in one of the two scanning positions. Further details are summarised in the ESM 1*.*

### Reference plane and orientation

Since multiple measurements were performed on each lesion, it was necessary to standardise the measurement process among the ten readers. To focus on potential size differences that are related to EC and window-level threshold, the orthogonal image plane that presented the largest diameter of a lesion was predefined. Furthermore, the orientation within the selected plane in which the calliper should be placed was proposed to the readers with a reference line indicating the largest diameter. The purpose of this approach was to reduce the possibility for biased results from intra- and inter-individual differences in the selection of the measurement plane and the measurement orientation. Details are summarised in the ESM 1.

### Reference size

The primary outcome measures were differences in the polyp size measurements of individual radiologists, related to EC within different CT-window settings. Therefore, the largest 2D linear diameter of a polyp, measured by each reader according to the recent guidelines proposed by the ESGAR in the unsubtracted CT dataset in a colon window (window, 1,500; level, -150) on the optimised image plane that best demonstrated this dimension, served as the reference polyp size [3]. To assess potential effects on polyp size that are related to EC and/or different CT-window settings, the polyp sizes measured within different EC and CT-window settings were compared to these reference size measurements. The rationale for this approach was to determine possible size differences related to EC and/or different CT-window settings for each reader, free from superimposing effects due to reported methodological inaccuracy or observer-associated variability, potentially associated with an OC, histopathological or expert CT reference size [12].

### Electronic cleansing algorithm

We used a commercially available software algorithm for electronic cleansing (Tagged stool subtraction; Siemens Healthineers, Erlangen, Germany). The basic function of the stool subtraction algorithm includes the following steps. Any voxel within the colon with a CT density value that exceeds a predefined dedicated threshold value, which is set at 100 HU, will be counted by the algorithm as potentially tagged faecal residue. Tagged residue with a density lower than 100 HU will not be recognised as stool and will not be electronically removed. Identified tagged residue with a CT density higher than 100 HU will then be digitally extracted from the datasets by attributing to them the density values of air. In this way, the colonic wall covered by faecal residue will become endoluminally visible in both 2D and 3D CTC images.

### Data evaluation

Data evaluation was performed on a commercially available server-based network of 3D workstations (Syngo.Via; Siemens Healthineers), with a standard CTC application (Syngo CT Colonography VB10) that is equipped with a commercially available software algorithm for electronic cleansing (Tagged stool subtraction).

All CTC data sets were interpreted independently by ten readers, all of them board-certified radiologists from two sites with moderate experience in CTC. The ten readers measured the size of each included colonic polyp six times; within three standardised CT-window/level settings (colon window 1,500, level -150; bone window 2,500, level 500; soft-tissue window 400, level 40), both before and after EC. The readers used a standard 2D measurement tool (manual calliper) that was available on the workstation. The measurements had to be performed in the predefined image slice along the orientation of the reference line representing the spatial orientation of the largest diameter of a lesion. The data were evaluated within six reading sessions in a fully randomised order within a time frame of 4 weeks. CT density values of tagged faecal residue surrounding the submerged polyps and the applied tube currents for each patient were recorded. Details on readers, measurements and randomisation are presented in the ESM 1*.*

### Statistical analysis

Descriptive statistics for each CT window were calculated for each of the ten readers, as well as averaged over the readers. Reader agreement was tested separately for each CT window and for measurements before and after EC, using intraclass correlation coefficients (ICCs). The reduction of the polyp size was calculated by subtracting the polyp size measure after EC from the measured polyp size before EC.

For statistical comparison, the polyp sizes, the reduction of the polyp sizes for different CT windows and the reduction of the polyp sizes due to EC were averaged over the ten readers. The Kolmogorov-Smirnov test was used to test for normal distribution. For normally distributed data, mean and standard deviations, as well as 95% confidence intervals, were used to describe polyp size and its reduction. In case of skewed data, the median and the first and third quartiles were used. Repeated measures ANOVA and the post hoc Bonferroni-corrected paired *t*-test were calculated to compare polyp size reduction for different CT windows. In order to test for a moderation effect of the polyp size category on differences between different CT windows, a MIXED model ANOVA with CT windows as a within-subject factor and initial polyp size groups as a between-subject factor were used.

Categorical data (e.g. downsizing of polyps into the next smaller size category (≥10 mm to <10 mm or ≥6 mm to <6 mm) were described, using absolute and relative frequencies and 95% confidence intervals. A value of *p* < 0.05 was considered to indicate statistical significance.

## Results

### Standard of reference

There were 37 patients (11 women, 26 men; age 50-76; mean age 59.08) with a total of 48 polyps ≥6 mm (33 small polyps, 6-9mm; 15 large polyps, ≥10mm; 34 adenomas, 14 non-adenomas) that were completely submerged within tagged faecal residue in at least one of both scanning positions who were included in the study (ESM Table S1*)*.

### Mean polyp size before and after EC

The mean polyp size before EC was significantly different within the three window settings, with 9.8 mm in the colon window, 9.9 mm in the bone window and 8.2 mm in a soft-tissue window (*p* < 0.001) (Table 1).

**Table 1:** Mean polyp size and the difference before and after electronic cleansing (EC) in a colon, bone and soft-tissue window. The differences for all window settings and all size groups are statistically significant (*p* < 0.001)

	Size groups	Before EC	After EC	Difference	Difference
	(mm)	(mm)	(mm)	(mm)	(%)
Colon window	6-9	7.0	6.7	-0.3	4.3
	≥10	15.8	15.3	-0.5	3.2
	≥6	9.8	9.4	-0.4	4.1
Bone window	6-9	7.2	6.3	-0.9	12.5
	≥10	15.9	15.1	-0.8	5.0
	≥6	9.9	9.1	-0.8	8.1
Soft-tissue window	6-9	5.2	4.0	-1.2	23.1
	≥10	14.8	13.7	-1.1	7.4
	≥6	8.2	7.1	-1.1	13.4

**Fig. 1:**
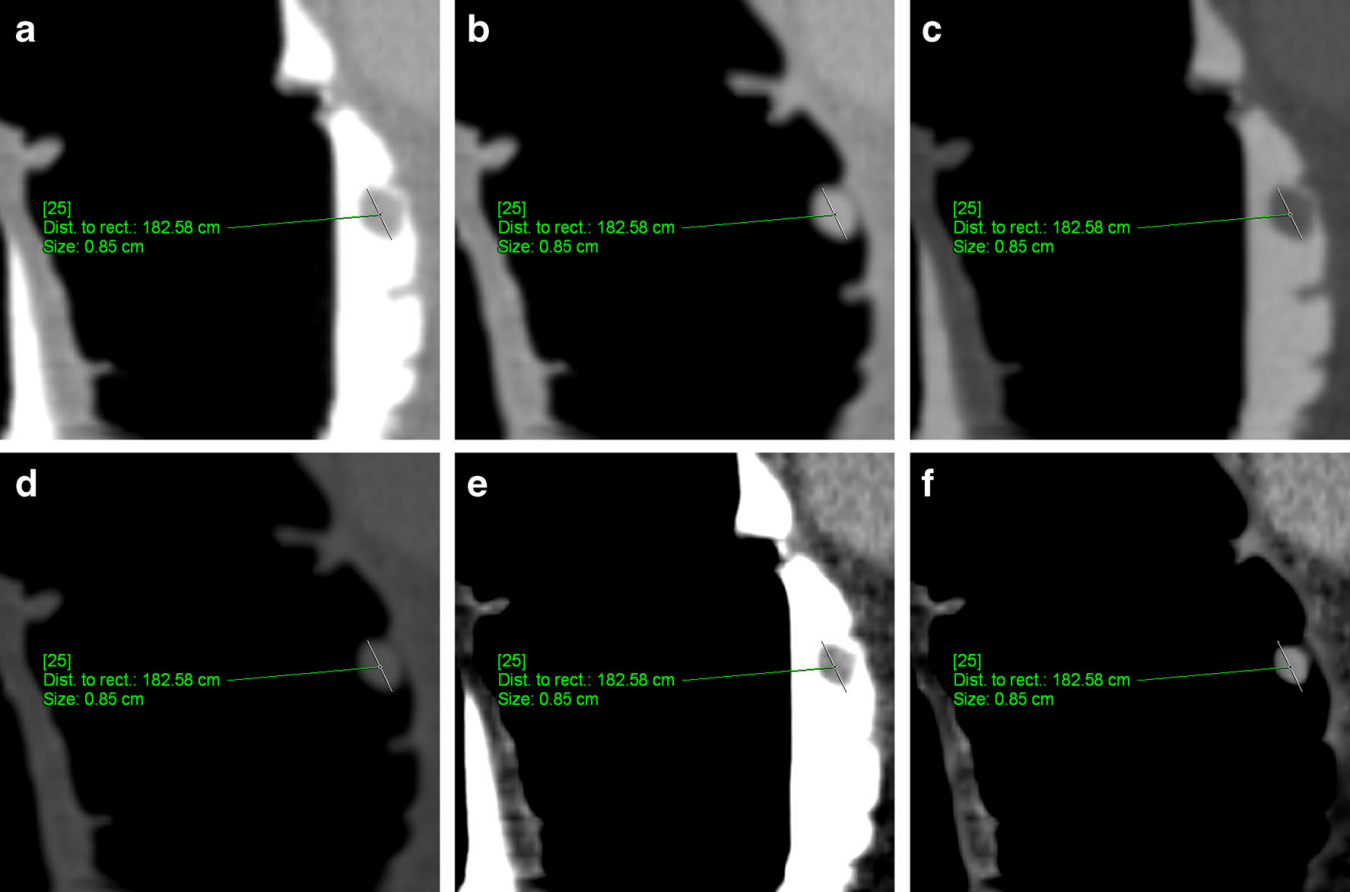
An 8.5-mm pedunculated polyp located in the ascending colon of a 71-year-old asymptomatic woman before and after EC. **a** The average size of the polyp measured by the ten readers was largest in the unsubtracted colon window (8.5 mm). **b** After EC, the average size of the polyps decreased to 8.3 mm. **c** In the unsubtracted bone window, the average polyp size was similar to that in the colon window (**d**)**,** decreasing to 7.8 mm after EC. **e, f** In the soft-tissue window, the average size of the polyps decreased to 7.0 mm before and 6.1 mm after EC

EC of tagged faecal residue led to a significant reduction of the mean polyp size by 0.4 mm in the colon window, 0.8 mm in the bone window and 1.1 mm in a soft-tissue window setting (*p* < 0.001). The size reduction was smallest in the colon window, compared with the bone and soft-tissue windows (*p* < 0.001 and *p* < 0.000). The mean polyp size after EC was 9.4 mm in colon, 9.1 mm in bone and 7.1 mm in a soft-tissue window setting. It was significantly smallest when measured in a soft-tissue window (*p* < 0.001) (Fig. 1).

The mean absolute size reduction of small and large polyps was in a comparable range for all windows: 0.3 mm and 0.5 mm, 0.9 mm and 0.8 mm, 1.2 mm and 1.1 mm for the colon, bone and soft-tissue windows, respectively. Conversely, the relative size reduction increased with decreasing polyp size (*p* < 0.001) with 4.3%, 12.5% and 23.1% for small and 3.2%, 5.0% and 7.4% for large polyps in a colon, bone and soft-tissue window setting, respectively.

### Influence of EC on polyp size categories

Changing the CT-window before EC from a colon to a bone window led to a shift from one small polyp (2.1%) to the group of diminutive lesions (<6 mm). In a soft-tissue window, however, 21 small polyps (43.8%) shifted into the group of diminutive polyps and three large polyps (20.0%) to the category of small polyps.

After EC, six small (12.5%) and one large polyp (6.7%) shifted to the next smaller size category in the colon window. In the bone window, nine small (19.1%) and one large polyp (6.7%)—and in the soft-tissue window, four small (14.8%) and two large polyps (16.7%)—were shifted into the next smaller size category (Table 2).

**Table 2:** Number of polyps in each size category and their differences before and after electronic cleansing (EC) in a colon, bone and a soft-tissue window, as well as compared to the unsubtracted colon window

	Groups	Before EC	After EC	Difference	Difference to
	(mm)	(*n*)	(*n*)	(*n*)	colon window (*n*)
Colon	≥10	15	14	-1	-1
window	≥6	48	42	-6	-6
Bone	≥10	15	14	-1	-1
window	≥6	47	38	-9	-10
Soft-tissue	≥10	12	10	-2	-5
window	≥6	27	23	-4	-25

**Fig. 2:**
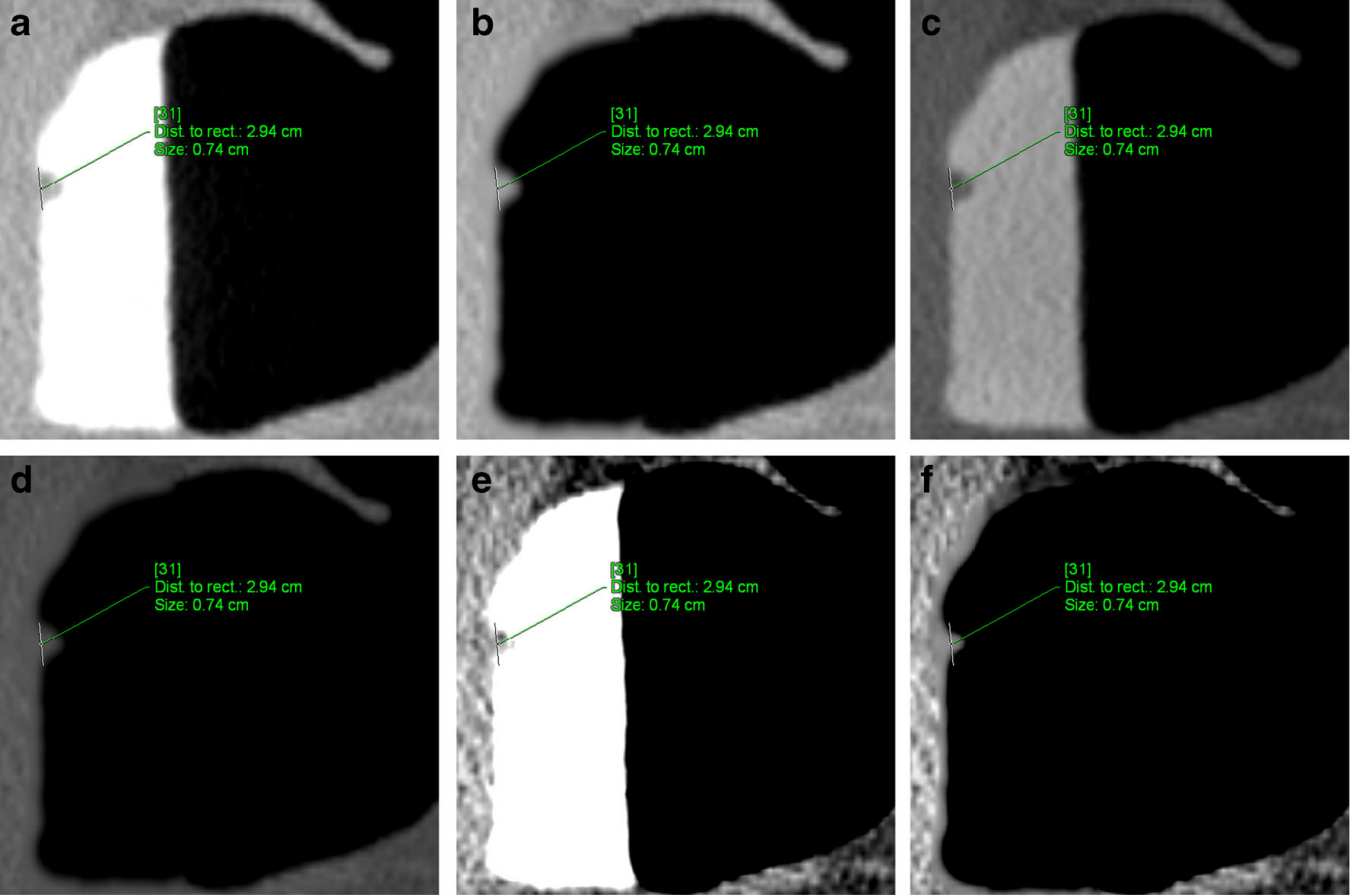
A 7.4-mm sessile polyp located in the rectum of a 57-year-old asymptomatic man before and after EC. **a** The average size of the polyp measured by the ten readers was largest in the unsubtracted colon window (7.4 mm). **b** After EC, the average size of the polyps decreased to 7.2 mm. **c** In the unsubtracted bone window, the average size of the polyps was similar to that in the colon window (**d**)**,** decreasing to 6.6 mm after EC. **e, f** In the soft-tissue window, the average size of the polyps decreased to 5.0 mm before and to 4.5 mm after EC, thereby causing a shift to the next smaller size category

The combination of changing the window setting and EC increased the shift of polyps to a smaller size category (Fig. 2). Compared to the unsubtracted colon window, EC performed in a bone window led to a shift of ten small polyps (20.8%) and one large polyp (6.7%) to the next smaller size category. In the soft-tissue window after EC, 25 of 48 polyps ≥6 mm (52.1%) were measured <6 mm and five of 15 polyps ≥10 mm (33.3%) were measured <10 mm.

### Results of individual readers

Intraclass correlation showed good agreement of the readers within the three CT windows before as well as after EC (Table 3).

**Table 3:** Intraclass correlation (ICC) with 95% confidence interval for each window setting before and after electronic cleansing (EC)

	95% confidence interval		
	ICC	Lower limit	Upper limit
Colon window before EC	0.996	0.994	0.998
Colon window after EC	0.994	0.992	0.997
Bone window before EC	0.993	0.989	0.996
Bone window after EC	0.989	0.983	0.993
Soft-tissue window before EC	0.993	0.990	0.996
Soft-tissue window after EC	0.990	0.983	0.994

The smallest average size reduction of polyps ≥6 mm related to EC was found for all readers in the colon window (0.1-0.5 mm; 1.1-5.8%). In the bone window, the differences in average polyp size reduction between individual readers were highest, ranging from 0.5 to 1.2 mm (5.3-11.7%). Within the soft-tissue window, eight of ten readers showed the largest polyp size reduction due to EC (1.0-1.3 mm; 11.6-16.2%) (Table 4).

**Table 4:** Mean difference in polyp size in millimetres and percentage due to electronic cleansing in the three window settings for each reader

Reader	Colon window		Bone window		Soft-tissue window	
	mm	%	mm	%	mm	%
1	-0.2	2.2	-0.6	5.8	-1.2	14.0
2	-0.4	3.6	-0.5	5.3	-1.2	13.9
3	-0.4	3.8	-0.8	7.7	-1.3	16.2
4	-0.3	3.5	-1.2	11.7	-1.1	14.2
5	-0.4	4.3	-0.7	6.7	-1.2	14.3
6	-0.5	5.8	-1.0	10.9	-1.1	13.5
7	-0.4	4.3	-0.8	7.9	-1.0	12.9
8	-0.5	5.6	-1.0	10.3	-1.1	13.7
9	-0.4	4.6	-1.1	11.4	-1.0	12.4
10	-0.1	1.1	-0.6	5.7	-1.0	11.6

### Effect of other parameters on EC

The mean CT density of tagged residue within the study population was 735 HU, ranging from 241 to 1,031 HU (SD, 215). There was no relationship between the reduction of polyp size and the CT density of tagged residue, the tube current and morphological and histological polyp subgroups (*p* > 0.05). The analysis is presented in detail in the ESM 1*.*

## Discussion

Little is known about the effect of EC on the morphological features of lesions submerged under faecal residue. From a clinical point of view, the most important morphological factor is polyp size. The 10-year risk for colorectal cancer increases with polyp size [13]. Due to lack of a pathohistological assessment, the polyp size represents the key criterion by which to estimate clinical relevance using CTC criteria [3]. Based on the recent European guidelines, there is general consensus that all polyps ≥6 mm need to be reported and endoscopic polypectomy should be recommended, while polyps <6 mm may be ignored [1, 3]. According to the CT Colonography Reporting and Data System (C-RADS), a categorisation scheme for colonic and extracolonic findings and for follow-up recommendations that may be applied for screening, patients with polyps 10 mm or larger require colonoscopy with resection (C3 category). However, for small lesions that measure between 6 and 9 mm, and if there are less than three in number (C2 category), CTC surveillance may be offered to the patient [14]. Therefore, the size of a polyp as measured by the radiologist has a direct influence on the selection of the appropriate therapeutic procedures [15].

We found that both EC and narrow window-level settings reduce significantly the size measurements of polyps. The size reduction was largest when EC was performed in soft-tissue windows and it was smallest using a colon window.

In addition, differences in window-level settings showed substantial influence on the size of submerged polyps, an observation that has already been previously described by Slater et al. [16]. The polyp size was largest when measured in wide window settings, such as colon or bone windows, and decreased when measured in soft-tissue windows, both before and after EC. Size differences of polyps in different window settings are believed to be related to the partial volume effect, which leads to a density gradient at the contrast media–polyp interface, with the window level and width defining how much of this gradient is included and, consequently, how much will be visible [17]. Size reductions after electronic cleansing most likely result from a slight transformation of the shape of the line attenuation profile of the polyps during the digital subtraction process.

Not surprisingly, the combination of both EC and the use of narrow window settings led to the largest decrease of polyp size. Compared to measurements taken in an unsubtracted colon window, which is generally agreed to display the largest diameter of the polyp most accurately [3], EC, performed in a soft-tissue window, led to a size reduction of 27.6% for polyps ≥6 mm and of 13.3% for polyps ≥10 mm.

Size reductions in polyps can be the source of misclassification of lesions into the wrong size category and, therefore, of underestimation of lesions of potential clinical relevance. Within each of the three window settings, EC led to a significant decrease in the number of polyps measuring ≥6 mm. However, when compared with the reference size measurements, taken in the unsubtracted colon window, the number of polyps shifting to smaller size categories increased further due to the additional size reduction related to CT-window changes, with 20.8% and 52.1% of submerged polyps ≥6 mm in bone and soft-tissue windows, respectively.

If being applied in the C-RADS system, EC would have led to a shift of 5, 7 and 17 patients from the C2 to the C1 category within colon, bone and soft-tissue windows, respectively, and 5 patients from the C3 to the C2 category in a soft-tissue window, when compared to the categorisation in an unsubtracted colon window.

While several studies have focused on the impact of EC on feasibility, sensitivity or interpretation time reductions [4, 5, 18–21], only one study has addressed the topic of a potential change in the size of submerged lesions [22]. Zalis et al. [22] evaluated in vitro the effect of EC software on size measurements of polyps in a colon phantom, repeatedly scanned without intraluminal contrast and with various bowel contrast material concentrations, a study design that cannot be applied in vivo. The mean measurement error of 0.6 mm assessed by two readers in 11 submerged polyps ≥8 mm was not significant at 200 and 500 HU, but increased to a significant level of 1.1 mm at 560 HU and 2.2 mm at 840 HU. Smaller lesions were not considered in this study.

Although not specifically investigated, other authors have also reported morphological effects on submerged lesions that were associated with EC. Serlie et al. [21] reported recognisable changes in the intraluminal appearance of polyps that were related to EC. Furthermore, the degeneration of bowel wall and colonic folds, as well as the distortion or degeneration of submerged polyps, were also found separately in different previous studies [4, 20]. Cai et al. [19] investigated an EC algorithm that potentially reduced EC-related artefacts due to incomplete subtraction at the air/contrast media interface. Strategies to further reduce EC-related artefacts and pitfalls may include the use of EC schemes that are based on the material decomposition capability of dual-energy CT, as shown in a recent phantom study [23, 24].

The results of this study show that measuring of submerged polyps after EC may account for shifting polyps to smaller size categories, a finding that is enhanced by using an inappropriate window setting. In the most unfavourable combination, when performing EC in a soft-tissue window setting, more than 50% of submerged lesions, measured as ≥6 mm, and 30% of submerged lesions ≥10 mm can be underestimated as <6 mm and as <10 mm, and therefore are either completely ignored or not resected.

This study has limitations. Some examiners may favour 3D over 2D measurements [25, 26]. It is, however, technically not possible to assess the influence of EC on 3D measurements because submerged polyps are surrounded by tagged residue, impairing the 3D reconstructions, which, however, are based on 2D image data. There has been expert agreement that the maximal diameter of lesions should be primarily estimated using 2D views, which were considered to be most reliable [3, 27]. Polyp size may vary within different 3D perspectives or appear larger due to contrast coating [18, 28].

A generally accepted “gold standard” for true polyp size would be desirable, since this would enable not only relative but also absolute size comparisons. However, endoscopic, histopathological or CT estimates are not wholly accurate and are affected by the way the measurement is obtained [3, 17, 29]. CT measurements, are, however, considered to provide a solid and reproducible estimate that is believed to range between endoscopic and pathohistological measurements [17, 30]. Finally, the decision for clinical treatment is solely based on CT measurements [17].

The presented results are limited to the applied algorithm for EC. It is possible that results may be different when using other algorithms for EC. However, since the basic principle of EC might also be applied in other approaches, it cannot be excluded that changes in lesion characteristics will also appear with other algorithms, as supported by observations in previous studies [4, 17, 19–21].

In conclusion, EC as well as narrow CT windows significantly reduce the size of polyps submerged in tagged residue. To avoid underestimation of polyp size, measurements of submerged polyps should be performed on unsubtracted image data in a colon-window setting.

## Electronic supplementary material

ESM 1 (DOCX 42 kb)

## **Abbreviations**

CTC: Computed tomography colonography
EC: Electronic cleansing
ESGAR: European Society of Gastrointestinal and Abdominal Radiology
ICC: Intraclass correlation coefficient
OC: Optical colonoscopy
