# Supplementary material for: CT colonography: size reduction of submerged colorectal polyps due to electronic cleansing and CT-window settings
Source: Eur Radiol. 2018 May 14;28(11):4766–74. doi: 10.1007/s00330-018-5416-0 (PMC6182748; doi:10.1007/s00330-018-5416-0)
Supplement: Supplementary file 1 — (DOCX 42 kb) [file 330_2018_5416_MOESM1_ESM.docx]

**Supplementary data to:**

# CT colonography: Size Reduction of Submerged Colorectal Polyps due to Electronic Cleansing and CT Window Settings

**Table of Contents:**

**Supplementary Material and Methods**

Sample size calculation and Power Analysis 2

Examination Technique 2

Reference Standard 3

Reference Plane and Orientation 4

Data Evaluation 4

Statistical Analysis 6

**Supplementary Results**

Supplementary Table 1: Patient and Polyp Distributions within the Reference Standard 7

**Supplementary Analysis**

Supplementary Analysis on potential relationships between CT density of tagged residue, tube current, and polyp histology and morphology with polyp size reduction related to EC 8

Supplementary Table 2 9

**References** 10

**Supplementary Material and Methods**

**Sample size calculation and power analysis**

The aim of the sample size calculation was to keep the limits of a two-sided 95% confidence interval for an average size reduction within +/- 0.5mm. Based on the results of a pilot study on a database of patients who were not included in this evaluation, a standard deviation of 1.1mm was expected.

A power calculation (NQuery Advisor 7.0) indicated that 19 polyps ≥6mm submerged in tagged fecal residue would be needed.

To assess whether EC results in shifting polyps from one polyp size category to another (<6mm, 6-9mm and ≥10mm), and assuming that the variance of the reduction in polyp size would be similar in both polyp size categories, the same polyp number was used for the ‘category 6-9mm’ and the category ‘≥10mm’. Thus, a total number of 38 polyps ≥6 mm, submerged in tagged fecal residue, were needed to power the study accordingly.

The low prevalence of colorectal adenomas ≥6mm in a screening population was recently reported, with 8.3 to 13.6% of patients with one or more adenomatous polyps ≥6mm [1; 2]. Assuming that 30% of polyps would be submerged under tagged fecal residue in at least one of the two scanning positions, a minimum number of 900 patients was required to identify these submerged polyps.

**Examination Technique**

CTC examination techniques for the included patient datasets were described previously in detail [1; 3; 4]. They were performed in adherence to published standards [5; 6]. In brief, after a clear liquid- or a low-residue diet, a standard 24-hour full laxative bowel preparation was applied [3; 4]. Fecal tagging was performed either with an iodine- and barium-based regimen [1; 3] or with an iodine-based regimen only [4]. Colonic distension was achieved using either manual insufflation of room air or automated CO_2_ insufflation. In 23 patients 20 mg of n-butyl scopolamine (Buscopan, Boeringer Ingelheim Pharmaceuticals, Ingelheim, Germany) were administered intravenously for bowel relaxation. All patients were scanned in the prone and supine positions, using 4- to 64-detector row scanners (GE LightSpeed Ultra, GE Medical Systems, Milwaukee, USA; Siemens Emotion 6 and Siemens Somatom Sensation 64, Siemens Healtheneers, Forchheim, Germany), with a reconstructed slice thickness of 0.75-1.25mm.

**Reference Standard**

Complete access to the reference data was used to perform a directed search in the CTC database to identify the patients with submerged colorectal lesions ≥6 mm, detected with CTC and OC.

This search was performed by two board-certified radiologists *(*T.M., P.L.) with 12 and 18 years of clinical and research experience, and consisted of more than 1900 and 10000 CTC cases on a commercially available CTC workstation (Syngo.Via, Siemens Healtheneers, Vitrea, Vital Images). The radiologists were assisted by a study coordinator (C.B.) with specific CTC research experience, responsible for data preparation and documentation. The data were not stratified with respect to examination quality, preparation quality, tagging, or degree of distention. Submerged polypoid findings ≥6mm that were seen only at CTC without a match to OC reports were not considered. Diminutive lesions (<6mm) were excluded from the analysis [5]. For each included lesion, the polyp size, morphology, and histology were recorded.

**Reference Plane and Orientation**

For each identified submerged polyp, the largest linear dimension of the polyp was assessed on two-dimensional axial or multiplanar CTC images, determined in a colon-window (window: 1500, level: -150), according to recent guidelines, on the CTC workstation [5]. The image plane (axial, coronal, sagittal) that showed the largest diameter of the polyp was identified and recorded. A reference line was placed along the planar orientation of the largest diameter of a polyp within this image plane. Definition of the measurement plane and orientation was determined by a board-certified radiologist (T.M.*),*assisted by the study coordinator, who was responsible for data preparation and documentation. The purpose of this approach was to guide the individual reader to perform multiple measurements for each lesion in the same plane in a comparable orientation, thus reducing the possibility for biased results from intra- and interindividual differences in the selection of the measurement plane and the measurement orientation.

**Data Evaluation**

***Readers***

The ten readers had been in radiological practice between seven and 17 years, and they had two to 10 years of clinical experience with CTC. The readers had previously attended a hands-on course in CTC, evaluating at least 50 validated CTC cases and/or they had practical experience in CTC during their radiological residency or routine clinical work.

The radiologists were blinded to the general aim of the study, the potential effects of digital stool subtraction, and/or different CT-window settings on polyp size, as well as the number and the size of included polyps. As a preparation for the evaluation, each reader was instructed about the specific workflow and the measurement process. The objective was to measure the largest diameter of colorectal polyps of at least 6mm in diameter.

***Randomization***

The data was evaluated by the 10 readers within six reading sessions in a fully randomized order within a time frame of four weeks. Within each reading session, every polyp was measured only once and randomly assigned to one of the six measurement conditions (colon-, bone-, and soft-tissue-window, both before and after EC) followed by another polyp, randomly assigned to one of the six measurement conditions. For each reading session, the order of the different polyps was fully randomized. The randomization of the order of the polyps was performed by associating a random number with each polyp and with each measurement condition. The random number was created by using the MS Excel RAND function (Microsoft Excel for Mac 2011, version 14.4.8, Microsoft). Then, all polyps where sorted after this random number to create a random order of polyps. The same was done for the measurement conditions of a polyp.

***Measurement Process***

For each reading session, the specific window/level settings, as well as the EC status, were prepared on the workstation by the study coordinator.

The predefined measurement plane was presented to the readers in a full-screen format, where the polyps were clearly perceptible. Readers were allowed to use the zoom function to enlarge the lesion, depending on their preferences. Furthermore, they were allowed to scroll through the adjacent images for better assessment of the 2D morphology and the dimensions of the lesion. They were not allowed to adjust the predefined window/level settings or the electronic cleansing setting. The readers used a standard 2D measurement tool (manual caliper) that was available on the workstation. The final measurements had to be performed in the predefined image slice along the orientation of the reference line representing the spatial orientation of the largest diameter of a lesion. The readers had to measure the largest diameter alongside this reference line, thus defining the lesion’s margin points with the manual caliper. The distance between these two margin points was defined as the size of the polyp.

After performing a measurement, the readers loaded up the next dataset for the measurement of another lesion within another measurement setting. All polyp measurements were recorded by the study coordinator. Before each reading session, the window/level settings and EC status were pre-adjusted by the study coordinator according to the randomization scheme.

**Statistical Analysis**

Statistical analysis was performed by an independent statistician (M.W.) who was not involved in the acquisition or computation of the data. The data were analyzed with the IBM SPSS statistical package (IBM SPSS Statistics for Windows, version 22.0; IBM).

**Supplementary Results**

**Supplementary Table 1:** Patient and Polyp Distributions within the Reference Standard

**Characteristics**  **n**

**Patients with submerged Polyps ≥ 6 mm**

Patients with at least one submerged Polyp

Overall 37

male 26

female 11

Distribution of submerged Polyps

Overall 48

male 32

female 16

Number of submerged polyps/patient

1 28

≥2 9

**Submerged Polyps ≥ 6 mm**

Size (mm)

≥ 6 mm 48

6-9 mm 33

≥ 10 mm 15

Morphologic characteristics

Sessile 21

Pedunculated 23

Flat 4

Histologic characteristics

Adenoma 34

Non-adenoma 14

Location

Right colon^1^ 21

Left colon^2^ 27

^1^cecum, ascending, and transverse colon

^2^descending, sigmoid colon, and rectum

**Supplementary Analysis**

**Supplementary Analysis on potential relationships between CT density of tagged residue, tube current, and polyp histology and morphology with polyp size reduction related to EC**

For each included lesion, the CT density values of the surrounding tagged fecal residue were measured by the study coordinator and recorded. This was done with the ROI (region of interest) tool, which was placed for each included lesion in the surrounding tagged residue close to the polyp within the measurement plane. In addition, the applied tube current (mAs) for each patient was recorded from the DICOM files.

To determine potential relationships between the reduction of the polyp size and the CT density values of the surrounding tagged fecal residue, as well as the tube current, Spearman’s correlation coefficients (rho) were calculated. Differences in polyp size reduction between morphologic and histologic subgroups were tested, using the Students t-test.

**Results**

The mean CT density of tagged residue within the study population was 735 H.U. ranging from 241 to 1031 H.U. (SD 215). Spearman’s correlation coefficients did not reveal any relationship between the reduction of polyp size and the CT density values of the surrounding tagged fecal residue, or the tube current. In addition, no significant differences in polyp size reduction were found between morphologic and histologic subgroups (P > .05) (Supplementary Table 2).

**Supplementary Table 2:** Correlation between absolute and relative reduction of polyp size due to electronic cleansing and the density values (H.U.) of the surrounding tagged fecal residue, as well as tube currents (mAs) at the CT section of the particular measurement in the three windows.

**Density Tube current**

**Reduction**  correlation coefficients -0.25 -0.24

**colon window** p-value 0.09 0.09

**Reduction**  correlation coefficients -0.13 -0.15

**bone window** p-value 0.39 0.30

**Reduction**  correlation coefficients -0.14 -0.16

**soft-tissue window** p-value 0.33 0.28

**Relative** **reduction** correlation coefficients -0.25 -0.28

**colon window** p-value 0.09 0.05

**Relative** **reduction** correlation coefficients -0.11 -0.18

**bone window** p-value 0.46 0.23

**Relative** **reduction** correlation coefficients -0.02 -0.23

**soft-tissue window** p-value 0.90 0.12

**References**

1 Lefere P, Silva C, Gryspeerdt S et al (2013) Teleradiology based CT colonography to screen a population group of a remote island; at average risk for colorectal cancer. Eur J Radiol 82:e262-267

2 Pickhardt PJ, Kim DH (2009) Colorectal cancer screening with CT colonography: key concepts regarding polyp prevalence, size, histology, morphology, and natural history. AJR Am J Roentgenol 193:40-46

3 Pickhardt PJ, Choi JR, Hwang I et al (2003) Computed tomographic virtual colonoscopy to screen for colorectal neoplasia in asymptomatic adults. N Engl J Med 349:2191-2200

4 Graser A, Stieber P, Nagel D et al (2009) Comparison of CT colonography, colonoscopy, sigmoidoscopy and faecal occult blood tests for the detection of advanced adenoma in an average risk population. Gut 58:241-248

5 Neri E, Halligan S, Hellstrom M et al (2013) The second ESGAR consensus statement on CT colonography. Eur Radiol 23:720-729

6 ACR–SAR–SCBT-MR Practice Parameter for the Performance of Computed Tomography (CT) Colonography in Adults. ACR practice guideline 2014. Available via https://<http://www.acr.org/Quality-Safety/Standards-Guidelines/Practice-Guidelines-by-Modality/CT>. Accessed June 9 2017
